# Endothelial progenitor cells are differentially impaired in ANCA-associated vasculitis compared to healthy controls

**DOI:** 10.1186/s13075-016-1044-8

**Published:** 2016-06-23

**Authors:** B. Wilde, A. Mertens, S. J. Arends, R. P. Rouhl, R. Bijleveld, J. Huitema, S. A. Timmermans, J. Damoiseaux, O. Witzke, A. M. Duijvestijn, P. van Paassen, R. J. van Oostenbrugge, J. W. Cohen Tervaert

**Affiliations:** Immunology, Maastricht University, PO Box 5800, 6202 AZ Maastricht, The Netherlands; Department of Nephrology, University Duisburg-Essen, University Hospital Essen, Essen, Germany; Department of Neurology, University Hospital Maastricht, Maastricht, The Netherlands; Central Diagnostic Laboratory, Maastricht University Medical Center, Maastricht, The Netherlands; Department of Infectious Diseases, University Duisburg-Essen, University Hospital Essen, Essen, Germany

**Keywords:** Endothelial progenitor cells, Autoantibodies, Vasculitis

## Abstract

**Background:**

Endothelial progenitor cells (EPC) are of major importance in vascular repair under healthy circumstances. Vascular injury in need of repair occurs frequently in ANCA-associated vasculitis (AAV). A specialized T cell subset enhancing EPC function and differentiation has recently been described. These angiogenic T cells (T_ang_) may have an important impact on the vascular repair process. Therefore, the aim of our study was to investigate EPC and T_ang_ in AAV.

**Methods:**

Fifty-three patients suffering from AAV and 29 healthy controls (HC) were enrolled in our study. Forty-four patients were in remission, nine patients were in active state of disease. Patients were either untreated or were under monotherapy with low-dose steroids (max. 5 mg/day) at the time of sampling. Circulating EPC and T_ang_ were determined by flow cytometry (FACS). The functional capacity of EPC was assessed by established cell culture methods.

**Results:**

Circulating EPC were significantly decreased in AAV as compared to HC. The capacity of EPC to differentiate and proliferate was differentially impaired in patients as compared to HC. The outgrowth of endothelial colony-forming cells (ECFC) was severely decreased in patients whereas colony-forming units-endothelial cell (CFU-EC) outgrowth was unaffected. ECFC and CFU-EC differentiation was strictly T cell-dependent. Patients with a relapsing disease course had an impaired ECFC outgrowth and expansion of T_ang_ as compared to patients with a stable, nonrelapsing disease.

**Conclusions:**

The differentiation process of EPC is impaired in AAV. This may favor insufficient vascular repair promoting a relapsing disease course. Finally, these factors may explain a higher cardiovascular morbidity as has been previously documented in AAV.

## Background

ANCA-associated vasculitis (AAV) is a small vessel vasculitis of autoimmune origin [1]. It is characterized by the presence of autoantibodies directed against either proteinase 3 (PR3) or myeloperoxidase (MPO) [1]. Treatment with immunosuppressive drugs reduces mortality significantly but needs to be continued even after remission has been achieved, and despite treatment, relapses occur frequently [2]. Persistent activation of clotting and the immune system – even in remission – indicates a “smoldering”, ongoing low-grade inflammation resulting in continuous vascular damage, which may contribute to the relapsing disease course [1, 3–5]. Accordingly, increased numbers of circulating endothelial cells, reflecting vascular damage, have been detected in patients in remission [6]. Importantly, AAV patients show increased cardiovascular morbidity and mortality [7–11].

In a healthy situation, vascular damage is repaired by so-called endothelial progenitor cells (EPC), which are mobilized from the bone marrow in situations when vascular injury occurs [12–14]. These EPC can be detected in the circulation. Furthermore, in vitro culture methods have revealed two different types of EPC. Endothelial colony-forming cells (ECFC) have been reported as the “real” progenitor cells replacing damaged endothelial cells whereas colony-forming units-endothelial cells (CFU-EC) have a supporting function providing the proper cytokine environment at the place of repair [14, 15]. Reduced EPC numbers, reflecting diminished vascular repair, are associated with cardiovascular morbidity and increased mortality [16]. Recent data suggests that also so-called angiogenic T cells (T_ang_) have a pivotal role in vascular repair and enhance EPC function [17].

We hypothesize that EPC are impaired in AAV, which is relevant for the relapsing disease course and the increased cardiovascular mortality. Therefore, we assessed EPC number by flow cytometry and function by means of in vitro culture assays. Furthermore, we investigated the role of angiogenic T cells, which are supposed to have a role in EPC differentiation and function, in our AAV patients.

## Methods

### Patient cohort

Fifty-three patients (mean age 58 ± 15 years) with diagnosed ANCA-associated vasculitis (Table 1) and 29 age-matched healthy volunteers were enrolled (mean age 58 ± 9 years). Nine patients had active disease at presentation and 44 AAV patients were in remission (Table 1). The diagnosis was made according to criteria of the American College of Rheumatology and the Chapel Hill Criteria [18, 19]. Active disease was defined as the presence of clinical manifestations of new-onset or recurrent disease activity related to vasculitis requiring intensified immunosuppressive therapy [20]. Remission/quiescent disease was defined as absence of clinical disease activity reflecting a Birmingham Vasculitis Activity Score of zero [20]. All patients included were either untreated or treated with a maximum dose of 5 mg steroids at the time of sampling. Current treatment with azathioprine, methotrexate, mycophenolate mofetil, cyclophosphamide or rituximab was an exclusion criterion. The cohort of patients in remission was divided in two groups for further analysis: (1) patients in long-term, stable remission (2) patients with relapsing disease course. Patients with a relapse-free disease course since the first onset of AAV and a minimum disease duration of 48 months were defined as being in stable long-term remission (nonrelapsers). Patients with a minimum disease duration of 48 months and at least one relapse within the first 48 months since diagnosis were defined as relapsers [21]. The study was approved by the local institutional review board and patients gave informed consent.

**Table 1:** Patients’ characteristics and demographics.

	AAV patients (n = 53)	
Age (yrs)	58 ± 15	
ANCA specificity		
	*PR3*	35 pts
	*MPO*	15 pts
	*None*	3 pts
Disease extent		
	*Limited AAV (no renal vasculitis)*	35 pts
	*Generalized AAV (with renal vasculitis)*	18 pts
Disease activity		
	*Remission*	44 pts
	*Mean CRP*	6.5 ± 5.1 mg/L
	*Active*	9 pts
	*Mean CRP*	79 ± 139 mg/L
	*Mean BVAS*	9 ± 6
Organ involvement in patients with active disease		
		*Number of patients*
	*Arthritis*	*2*
	*Ear-nose-throat*	*3*
	*Ophthamologic*	*2*
	*Kidney*	*3*
	*Lung*	*3*
	*Serositis*	*1*

*AAV* ANCA-associated vasculitis, *BVAS* Birmingham Vasculitis Activity Score, *CRP* C-reactive protein, *MPO* myeloperoxidase, *PR3* proteinase 3

### Flow cytometric analysis

Circulating endothelial progenitor cells (cEPC) and T cells were determined by flow cytometry (FACS). The following antibodies labeled with fluorescent dye were used: α-human CD3 (mouse IgG1, HorV450), α-human CD4 (mouse IgG1, PerCP; ITK/Biolegend, Uithoorn, The Netherlands), α-human CD28 (mouse IgG1, APC), α-human CD31 (mouse IgG1, FITC), α-human CD34 (mouse IgG1, PerCP), α-human CD45 (mouse IgG1, AmCyan), α-human KDR/VEGFR2 (mouse IgG1, PerCP, R&D Systems, Oxon, United Kingdom), α-human CD133/1 (mouse IgG1, APC, Miltenyi Biotec, Leiden, The Netherlands). All antibodies except CD4, KDR, and CD133 were purchased from BD Biosciences, Breda, The Netherlands. Appropriate isotype controls (BD Biosciences) were used. In addition, an unstained control sample tube was included to control for autofluorescence. Monoclonal antibodies were added to 150 μL of whole blood followed by an incubation period of 30 min in the dark at room temperature. After red blood cell lysis, at least 100,000 events were acquired in the lymphocyte gate by FACS. Sample carryover was minimized by automatic rinsing of the acquisition needle after each sample tube. Furthermore, a minimum of 100,000 lymphocytes were acquired per sample tube to ensure accuracy of the measurement. The protocol was adopted and modified after Duda et al. [22]. Analysis was performed with a FACS CANTO™ from BD Biosciences.

Data was analyzed using FACS DIVA software (BD Biosciences). Angiogenic T cells were defined as CD3^+^CD31^+^ according to the definition by Hur et al [17]. cEPC were defined as CD45^dim^/CD34^+^/KDR^+^ cells [23]. Absolute cell counts were calculated from complete blood counts.

### Determination of soluble interleukin-2 receptor and neopterin plasma levels

Soluble interleukin-2 receptor (sIL-2r) and neopterin levels were determined in plasma samples by commercially available ELISA kits (sIL-2r: Diaclone Research, Besancon, France; neopterin: IBL International, Hamburg, Germany) according to the manufacturer’s instructions.

### In vitro culture of endothelial progenitor cells

Peripheral blood mononuclear cells (PBMC) were obtained and cultured as previously described [24, 25]. PBMCs were isolated by standard Ficoll density gradient centrifugation. Subsequently, the recovered cells were washed with PBS and resuspended in HUVEC culture medium containing RPMI 1640 (Glutamax, GIBCO/Invitrogen, Breda, The Netherlands), 20 % fetal calf serum (FCS, Integro, Lelystad, The Netherlands), 100 IU/mL penicillin and 100 μg/mL streptomycin (Gibco), heparin (20 IE/mL; Leo Pharma, Breda, The Netherlands) and endothelial cell growth factor (Reliatech, Wolfenbüttel, Germany) at a concentration of 4*10^6^/mL. 4*10^6^/well were seeded in a 24-well plate, which was coated before with gelatin 1 %. The cells were cultured for the next 48 hours at 37 °C, 5 % CO2. Forty-eight hours after incubation, nonadherent cells were collected and seeded at a concentration of 1*10^6^/mL on a gelatine-coated 24-well plate. These cells were cultured for another 5 days to obtain CFU-EC type EPC [24, 25]. Moreover, the remaining adherent cells were also cultured for another 5 days to obtain ECFC-type EPC [24, 25].

The amounts of clusters were counted before refreshing the media in both the ECFC- and the CFU-EC wells. A cluster consisted of a colony of multiple (>20) thin cobblestone-shaped or spindle-shaped cells, tightly clustered together. Clusters were counted manually in at least two wells per condition by two independent observers [24, 25]. The calculated intra-assay coefficient of variation was 21 %.

### Statistical analysis

Data are expressed as mean ± SD or median (interquartile range, IQR). The lower and upper 95 % confidence interval of the mean is given in addition (CI). Statistical significance was tested by applying a two-tailed Mann-Whitney *U* test. Correlation analysis was performed by means of the Spearman’s rank correlation. All statistical analyses were performed with the use of GraphPad Prism software, version 5.03 (GraphPad Software, Inc., La Jolla, CA, USA). A *p* value < 0.05 was considered as statistically significant.

## Results

### Reduced numbers of cEPC are accompanied by defective outgrowth of ECFC

**Fig. 1:**
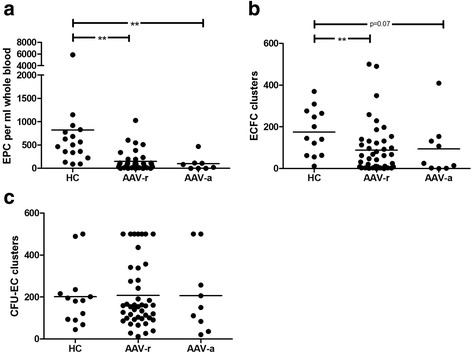
Endothelial progenitor cells are diminished and differentially impaired in patients with ANCA-associated vasculitis (AAV). **a** Circulating endothelial progenitor cells (EPC) are diminished in patients with active or inactive disease as compared to HC. **b**, **c** In patients, proliferative capacity of EPC of the endothelial cell-forming colonies (ECFC) type is impaired whereas colony-forming units-endothelial cells (CFU-EC) EPC are unaffected. *Horizontal bars* represent the mean value. **p value < 0.005. *AAV-a* AAV patients with active disease, *AAV-r* patients in remission, *HC* healthy controls

cEPC (CD45^dim^/CD34^+^/KDR^+^) were determined directly ex vivo by flow cytometry. There was a decrease of cEPC in patients with AAV in remission (AAV-r) and patients with active AAV (AAV-a) as compared to healthy controls (HC) (given as cells/mL whole blood: AAV-r: 75 (IQR 156, CI 75–220) vs. 504 (IQR 521, CI 139–1508), *p* < 0.0001; AAV-a: 52 (IQR 112, CI -32 to 231) vs. 504 (IQR 521, CI 139–1508), *p* = 0.001, Fig. 1a). No differences were detected between AAV-r and AAV-a (Fig. 1a). Proliferative capacity of EPC was tested in a culture assay allowing distinguishing between ECFC and CFU-EC. The number of ECFC clusters was diminished in quiescent and active patients as compared to HC (given as clusters/well: 45 (IQR 125, CI 51–125) vs. 145 (IQR 208, CI 107–242), *p* < 0.005 and 25 (IQR 141, CI -9 to 196) vs. 145 (IQR 208, CI 107–242), *p* = 0.07, Fig. 1b). Outgrowth of CFU-EC, however, was unaltered in patients as compared to HC (Fig. 1c).

**Fig. 2:**
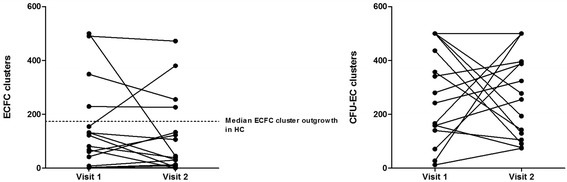
Longitudinal assessment of EPC differentiation in AAV patients. **a** ECFC outgrowth was rather stable over time and did not normalize. **b** CFU-EC outgrowth was fluctuating over time. *CFU-EC* colony-forming units-endothelial cells, *ECFC* endothelial cell-forming colonies, *HC* healthy controls

When assessed longitudinally, ECFC outgrowth was relatively stable in AAV patients and did not normalize over time (Fig. 2a). However, CFU-EC outgrowth was less stable (Fig. 2b).

Thus, reduced numbers of cEPC in patients seem to be accompanied by a specific impairment of ECFC whereas CFU-EC seem intact.

### Angiogenic T cells impact ECFC and CFU-EC differentiation

**Fig. 3:**
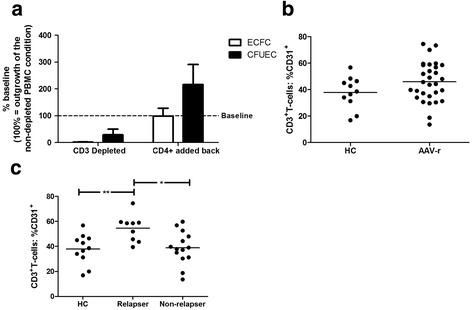
Critical role of T cells in ECFC and CFU-EC differentiation. **a** Depletion of T cells from ECFC or CFU-EC cultures abolished EPC differentiation. Adding back T cells restored ECFC and CFU-EC outgrowth indicating the pivotal role of T cells in promoting EPC differentiation. **b** No significant difference was found between HC and AAV-r regarding the fraction of circulating CD31^+^ T cells. **c** Stratifying patients according to clinical course reveals that frequent relapsers have an expanded compartment of circulating angiogenic T cells as compared to HC or patients with nonrelapsing disease course. *Horizontal bars* represent the mean value, *error bars* represent the standard of the mean. *p = 0.01, **p = 0.006. *AAV-r* patients in remission, *CFU-EC* colony-forming units-endothelial cells, *ECFC* endothelial cell-forming colonies, *HC* healthy controls, *PBMC* peripheral blood mononuclear cells

T cells have been described to control and support EPC function [17]. T cells (CD3^+^) were depleted from PBMC and cultured. As expected, T cell depletion abolished ECFC and CFU-EC outgrowth (Fig. 3a). Adding back T helper cells to T cell-depleted PBMC cultures recovered ECFC and CFU-EC differentiation confirming the essential role of T cells for EPC differentiation. Therefore, angiogenic T cells (CD3^+^CD31^+^, T_ang_) were further studied in AAV patients.

We found no difference in relative or absolute numbers of T_ang_ when comparing patients in remission and HC (% of CD3^+^ T cells: 45 (IQR 24, CI 40–52) vs. 39 (IQR 15, CI 30–46), *p* = 0.15; T_ang_/μL whole blood: 398 (IQR 324, CI 364–620) vs. 423 (IQR 298, CI 293–508), *p* = 0.74, Fig. 3b). The degree of inflammation (reflected by sIL-2R or neopterin serum levels), however, correlated negatively with absolute T_ang_ numbers (r = -0.52, *p* = 0.01, n = 23 and r = -0.43, *p* = 0.04, n = 23) in quiescent AAV.

### Relapsing disease course is associated with impaired ECFC outgrowth

**Fig. 4:**
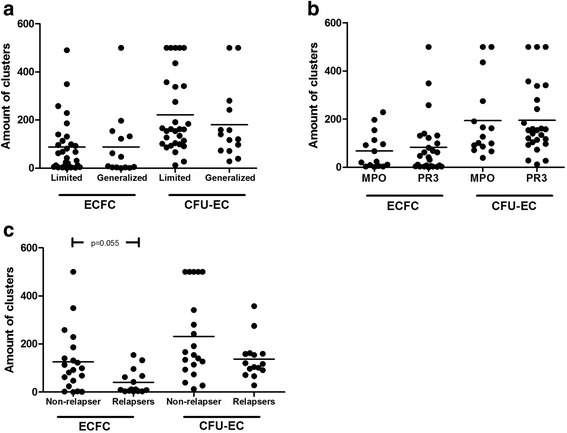
Patients with relapsing disease course have a more profound impaired proliferative capacity of ECFC when compared to patients with stable, nonrelapsing disease course. **a**, **b** Stratifying the patient cohort according to disease extent – limited versus generalized AAV- or type of autoantibody – anti-MPO versus anti-PR3- did not show any differences with regard to proliferative capacity of ECFC/CFU-EC-type EPC. **c** In patients with relapsing disease course, the proliferative capacity of ECFC is more impaired than in patients with stable disease*. Horizontal bars* represent the mean value. *CFU-EC* colony-forming units-endothelial cells, *ECFC* endothelial cell-forming colonies, *MPO* myeloperoxidase, *PR3* proteinase 3

Subgroup analyses were performed to assess clinical associations linked to reduced cEPC or diminished ECFC outgrowth. Stratification by renal involvement did not show significant differences between the groups (Fig. 4a). Likewise, stratifying the patient cohort according to ANCA-type (PR3/MPO) revealed no significant differences (Fig. 4b). There was no association between ECFC and serum creatinine, platelet count, C-reactive protein or leukocytes (r = -0.29, 0.14, r = -0.04 and r = 0.16, all *p* > 0.05). CFU-EC correlated weakly with leukocyte count (r = -0.35, *p* = 0.02) but not with serum creatinine, platelet count or C-reactive protein (r = -0.05, r = 0.18 and r = -0.1, all *p* > 0.05). cEPC were not significantly associated with any of the parameters (data not shown). Furthermore, neither siL2r nor neopterin correlated with ECFC, CFU-EC, or cEPC (r = -0.27, r = -0.008; r = -0.21, r = -0.19; r = 0.28, r = 0.26; all *p* value > 0.05). However, a relapsing disease course was specifically associated with impaired ECFC outgrowth but not with CFU-EC outgrowth (Fig. 4c) or cEPC numbers. Moreover, patients with a relapsing disease course showed an expansion of T_ang_ whereas patients in long-term stable remission were not different from HC (% of CD3^+^ T cells: 59 (IQR 15, 46–63) vs. 39 (IQR 15, CI 30–46), *p* = 0.006 and 39 (IQR 18, CI 31–47) vs. 39 (IQR 15, CI 30–46), *p* = 0.89; Fig. 3c).

## Discussion

In this study, we demonstrated that AAV patients have lower numbers of circulating EPC and we showed that differentiation into endothelial colony-forming cells (ECFC) is impaired as compared to HC. Furthermore, we found that a relapsing disease course in AAV is associated with a reduced outgrowth of ECFC. Finally, we confirmed that T cells are essential for EPC differentiation.

Previously, it has been reported that AAV patients have diminished numbers of circulating EPC [26–28]. In our study, we confirmed that cEPC were diminished in AAV. Also, we found that ECFC derived from AAV patients had an impaired proliferative capacity indicating that the repair process of damaged endothelium might be impaired in AAV. Woywodt et al. demonstrated that AAV patients with quiescent disease have persistently increased numbers of circulating endothelial cells which may be released from sites of vascular damage [6]. Based on these observations, we postulate that even in quiescent disease there is continuous vascular injury which needs to be repaired. The overall decrease and reduced differentiation capacity of EPC populations may cause insufficient vascular repair. This may contribute to the higher cardiovascular morbidity and mortality in AAV patients [8].

Because vascular repair is delayed, endothelial damage may persist resulting in a stimulation of the immune system [29]. Continuous vascular injury may then favor chronic inflammatory immune responses promoting disease relapses [1, 3, 29].This might explain why patients with a relapsing disease course had the lowest ECFC numbers in comparison to patients with a nonrelapsing disease course or HC.

The results of our study differed from previous studies [25–27]. In an earlier study by de Groot et al, AAV patients and HC showed no difference in hematopoietic stem cell numbers [26]. Furthermore, in this study with 11 active patients, no significant differences between age-matched HC and AAV-a were found with respect to ECFC. The differences between this study and our study might be explained by different techniques used to detect and quantify ECFC. In contrast to our assay, nonadherent cells were removed later from the ECFC culture; furthermore, ECFC were re-seeded within the first week of culture. The timing of seeding and removal of nonadherent cells is critical to the outgrowth of ECFC as has been described before [30, 31]. Zavada et al. found CFU-EC to be diminished in patients with AAV as compared to HC [27]. This is in contrast to the findings in our study where we found the outgrowth of CFU-EC to be comparable in HC and in AAV patients. Differences in the patient cohort probably explain these different findings. Most of the patients in remission were treated with immunosuppressive drugs (93 %) in the Zavada study, whereas in our study patients in remission were only eligible if left untreated or receiving a maximum 5 mg of prednisone. Interestingly, another study by Zavada et al. demonstrated an association of CFU-EC numbers and relapse rate [28]. In our study, we found an association of ECFC, but not CFU-EC, outgrowth, and tendency to relapse. Again, this discrepancy might be explained by the cohort characteristics; the patients enrolled by Zavada et al. were mostly treated with immunosuppressive agents.

Only recently, a pro-angiogenic role has been described for T cells [17, 25]. In our study, we could confirm that T cells are essential for EPC differentiation. T_ang_, which are suggested to have pro-angiogenic function, however, have never been studied in AAV before. We found no difference comparing T_ang_ numbers between AAV patients and HC. Subgroup analysis revealed that AAV patients with relapsing disease course showed an expansion of T_ang_ whereas ECFC numbers were lower in these patients. T_ang_ expansion may be an ineffective attempt to compensate the need for increased EPC function.

In summary, we provide evidence that EPC are impaired in AAV. We postulate that insufficient vascular repair combined with chronic vascular injury may increase the relapse propensity. These factors may contribute to the higher cardiovascular morbidity as previously documented in AAV.

## Conclusions

We demonstrate that in patients with ANCA-associated vasculitis (AAV) endothelial progenitor cells are differentially impaired as compared to healthy controls: endothelial colony-forming cells (ECFC-type EPC) show defective differentiation capacity, especially in patients with relapsing disease course, whereas colony-forming units-endothelial cells (CFU-EC-type EPC) are intact. Furthermore, we confirmed that T cells are essential for ECFC/CFU-EC differentiation and found that these angiogenic T cells are abnormal in relapsing AAV. In summary, insufficient vascular repair in AAV may favor a relapsing disease course. Finally, these factors may explain a higher cardiovascular morbidity as has been previously documented in AAV.

## Abbreviations

AAV, ANCA-associated vasculitis; AAV-a, AAV patients with active disease; AAV-r, AAV patients in remission; CI, confidence interval; CFU-EC, colony-forming units-endothelial cells; ECFC, endothelial colony-forming cells; EPC, endothelial progenitor cells; cEPC, circulating endothelial progenitor cells; HC, healthy controls; IQR, interquartile range; MPO, myeloperoxidase; PBMC, peripheral blood mononuclear cells; PR3, proteinase 3; sIL-2r, soluble interleukin-2 receptor; T_ang_, angiogenic T cells.
